# Saliva stress biomarkers in ERCP trainees before and after familiarisation with ERCP on a virtual simulator

**DOI:** 10.3389/fsurg.2024.1364195

**Published:** 2024-06-17

**Authors:** Konstantinos Georgiou, Nikola Boyanov, Dimitrios Thanasas, Gabriel Sandblom, Dimitrios Linardoutsos, Lars Enochsson

**Affiliations:** ^1^ 1st Department of Propaedeutic Surgery, Hippocrateion Athens General Hospital, Athens Medical School, National and Kapodistrian University of Athens, Athens, Greece; ^2^ Medical Simulation Training Centre, Research Institute of Medical University of Plovdiv, Plovdiv, Bulgaria; ^3^ Medical Physics Laboratory Simulation Centre, Athens Medical School, National and Kapodistrian University of Athens, Athens, Greece; ^4^ Department of Clinical Science and Education Södersjukhuset, Karolinska Institute, Stockholm, Sweden; ^5^ Department of Surgery, Södersjukhuset, Stockholm, Sweden; ^6^ Department of Diagnostics and Intervention, Surgery, Umeå University, Umeå, Sweden; ^7^ Department of Clinical Science, Interventions and Technology, Division of Orthopedics and Biotechnology, Karolinska Institutet, Stockholm, Sweden.

**Keywords:** ERCP, virtual simulator, training, stress, saliva biomarkers

## Abstract

**Background:**

Stress during the early ERCP learning curve may interfere with acquisition of skills during training. The purpose of this study was to compare stress biomarkers in the saliva of trainees before and after familiarisation with ERCP exercises on a virtual simulator.

**Methods:**

Altogether 26 endoscopists under training, 14 women and 12 men, completed the three phases of this study: Phase 1. Three different ERCP procedures were performed on the simulator. Saliva for α-amylase (sAA), Chromogranin A (sCgA), and Cortisol (sC) were collected before (baseline), halfway through the exercise (ex.), and 10 min after completion of the exercise (comp.); Phase 2. A three-week familiarisation period where at least 30 different cases were performed on the virtual ERCP simulator; and Phase 3. Identical to Phase 1 where saliva samples were once again collected at baseline, during, and after the exercise. Percentage differences in biomarker levels between baseline and exercise (Diff_ex_) and between baseline and completion (Diff_comp_) during Phase 1 and Phase 3 were calculated for each stress marker.

**Results:**

Mean % changes, Diff_ex_ and Diff_comp_, were significantly positive (*p* < 0.05) for all markers in both Phase 1 and Phase 3. Diff_ex_ in Phase 1 was significantly greater than Diff_ex_ in Phase 3 (*p* < 0.05) for sAA and sCgA. Diff_comp_ for sAA in Phase 1 was significantly greater than Diff_comp_ in Phase 3 (*p* < 0.05). No significant differences were found in sC concentration between Phases 1 and 3.

**Conclusion:**

This study shows that familiarisation with the ERCP simulator greatly reduced stress as measured by the three saliva stress biomarkers used with sAA being the best. It also suggests that familiarisation with an ERCP simulator might reduce stress in the clinical setting.

## 1 Introduction

ERCP is a technically demanding endoscopic procedure requiring a high level of expertise to provide effective and safe patient care. Somehow, trainees must be able to practice effectively without putting the safety of the patient in jeopardy. Simulator-based training is thus highly recommended (1).

Virtual reality simulation is an established method for acquiring and improving technical and non-technical skills in a controlled, reproducible, and quantitative environment that replicates real psychological challenges and mental stress. Simulation training has been used to assess stress and to develop intraprocedural stress management (2). The complexity of stress mechanisms makes acute stress measurement difficult to quantify and interpret. There is no universally recognised non-invasive gold standard technique for the assessment of stress. Instead, subjective, and objective surrogate methods have been proposed, such as measuring acute changes in the autonomic nervous system (ANS). Furthermore, non-invasive measurement of actual stress during a clinical procedure is not practically feasible (3).

There are objective biomarkers of stress in the blood and saliva. The use of blood biomarkers involves the invasive procedure of taking blood, whereas saliva sampling is relatively non-invasive and thus a better way to analyse stress. According to the literature, the most effective salivary biomarkers of stress are cortisol (sC), alpha-amylase (sAA), and chromogranin A (sCgA) (4). Since stress is not dichotomous there are no specific thresholds for these biomarkers that indicate a high level of stress (5). Furthermore, stress is usually associated with impaired performance which, in the clinical situation, could lead to complications. Moreover, it has been shown that trainees suffer greater stress than more experienced practitioners (6).

When training individuals in colonoscopy, steeper learning curves and fewer complications have been observed when employing a simulator-based program in training (7). These benefits have yet to be shown for ERCP (8). Furthermore, in a recent systematic review on the use of simulators to acquire ERCP skills, only one study conformed with validation criteria (9).

Our primary objective was thus to non-invasively measure stress levels of ERCP trainees by means of saliva stress biomarkers before and after familiarisation with virtual reality (VR) ERCP simulation. A secondary outcome was to assess any differences in stress between men and women.

## 2 Materials and methods

### 2.1 Participants

Fifty-one trainee residents aged 28–34 years were enrolled. All participants were residents in gastroenterology and general surgery, without any previous experience in ERCP. Written informed consent was obtained from each participant. The experiments were performed at the Medical Simulation Training Centre at the Medical University, Plovdiv, Bulgaria.

### 2.2 Experiments procedure

#### 2.2.1 Initial exercise: phase 1

All subjects answered a baseline questionnaire for information on demographic data and prior endoscopic or simulator experience. They conformed with the following inclusion criteria: (1) no current prescribed or non-prescription medication; (2) no flu or symptom of upper respiratory tract infection; (3) non-smoker; (4) no alcohol, coffee, or exercise within 12 h prior to testing; and (5) no food or brushing of teeth within 1 h of the experiment. A post-experiment questionnaire regarding the participant's perception of the project was answered.

After a rest period of 30 min, a baseline saliva sample was collected using an unstimulated passive drool technique. The subjects then spent 30 min getting used to the ERCP modules on the GI-Mentor II simulator (Surgical Science Sweden AB, Gothenburg, Sweden) as well as add-on software (guidewires *etc*). Then they watched a video prepared by the second author, demonstrating the three ERCP exercises to be performed in the hands-on session. They then performed virtual bile duct cannulation three times to become acquainted with the simulator. The participants then completed, to the best of their ability, the following three virtual ERCP exercises with increasing level of difficulty:

a. *ERCP Exercise 1: Bile duct stone removal (ERCP Module 1, Case Study 4)*.

Deep cannulation of the bile duct (BD) with a sphincterotomy catheter, contrast injection to diagnose the common bile duct stone (CBDS), then sphincterotomy followed by stone extraction using an extraction balloon.

b. *ERCP Exercise 2: Diagnosis of hilar stenosis, and brush cytology (ERCP Module 1, Case Study 2)*.

Cannulation of the BD and insertion of a guidewire. Contrast injection to reveal hilar stenosis. After sphincterotomy, brush cytology of the stenosis is performed.

c. *ERCP Exercise 3: Diagnosis of cystic bile duct leakage and treatment with placement of a bile duct stent (ERCP Module 2, Case Study 4)*.

BD cannulation using a sphincterotomy catheter followed by contrast injection revealing cystic leakage. After sphincterotomy, a plastic stent is introduced to cover the site of the cystic duct leakage.

No mentor intervention was allowed during the exercise session. Halfway through the first exercise (Phase 1), an “exercise” saliva sample was collected, and 10 min after completing the exercise a third “completion” saliva specimen was taken as before. The saliva samples were refrigerated and subsequently stored at −20 °C within 4 h of collection pending analysis. Participants unable to complete the three ERCP exercises of Phase 1 were excluded as no comparison with the reciprocal exercises of Phase 3 could be achieved. Therefore, only the remaining 26 proceeded on to Phase 2.

#### 2.2.2 Familiarisation: phase 2

During the following three weeks, the participants remaining became familiar with the ERCP simulator by performing at will at least 30 procedures supervised by a mentor (not including the initial three exercise procedures in Phase 1).

#### 2.2.3 Repetition: phase 3

After familiarisation, the remaining participants completed the three exercises exactly as in Phase 1 described above, and new saliva specimens (baseline, exercise, and completion) were collected and stored for analysis.

### 2.3 Data analysis

All saliva samples were assessed using commercially available kits. For salivary cortisol (SME-1- 3002 Salivary Cortisol Research ELISA kit) and for *α*-amylase (SME-1- 1902 Alpha-amylase Kinetic Reaction Kit Research) were used, both from Salimetrics, Carlsbad, CA, USA (www.salimetrics.com). Human Chromogranin A (sCgA) was measured with an EIA Kit (Cat. No.: RSCYK070R, BioVendor GmbH Germany). Concentrations of the saliva biomarkers were determined following the manufacturer's instructions.

To reduce multifactorial stress bias from external factors, the percentage difference (Diff_ex_) of each saliva biomarker was calculated from its baseline and exercise values, as well as the corresponding difference (Diff_comp_) between baseline and completion values. Accordingly, Diff_ex _= 100 × (Value_ex_−Value_bas_)/Value_bas_ and Diff_comp_ = 100 × (Value_comp_−Value_bas_)/Value_bas_.

### 2.4 Statistical analysis

The normality of the collected data was tested using the Kolmogorov–Smirnov test. The R software version 3.5.0 was used for statistical analysis (10).

We used paired *t*-test to compare mean Diff_ex_ between Phases 1 and 3 as well as between Diff_comp_ between Phases 1 and 3. We also used one-sided t- test to determine whether mean Diff_ex_ or Diff_comp_ in Phases 1 and 3 were positive showing that saliva parameter values during exercise and completion were significantly greater than their corresponding baseline.

## 3 Results

Twenty-five of the 51 participants were unable to complete Phase 1 and were therefore excluded from the study. Thus, our final group consisted of 26 participants (12 men and 14 women), 7 were 20–30 years of age and the remaining (*n* = 19) 30–40 years of age.

All participants experienced saliva collection to be problem-free and did not cause distraction. The distribution of the collected data was tested using the Kolmogorov–Smirnov test and found to be normal.

Diff_ex_ and Diff_comp_ values were calculated from the Phase 1 and Phase 3 saliva biomarker data. Thus, six percentage differences Diff_ex_ and Diff_comp_ for the three saliva biomarkers were estimated in Phase 1 and six in Phase 3 (see Tables 1–3).

**Table 1:** Diff_ex_(%) and diff_comp_(%) for saliva biomarkers (mean + SD) in phases 1 and 3.

a-amylase	Phase 1		Phase 2		*p*-value
	Mean	SD	Mean	SD	
Diff_ex_	185	457.3	8.6	53.2	<0.05
Diff_comp_	264	585.3	28.7	83.1	<0.05
Chromogranin A					
Diff_ex_	71	97.7	42.4	49.7	<0.05
Diff_comp_	47.9	81.3	32.9	41.4	0.23
Cortisol					
Diff_ex_	31.4	32.3	22.5	33.7	0.18
Diff_comp_	46.4	36	42	51.4	0.36

**Table 2:** Male group (*n* = 12): diff_ex_(%) and diff_comp_(%) (mean + SD) for each saliva biomarker in phases 1 and 3.

a-amylase	Phase 1		Phase 3		*p*-value
	Mean	SD	Mean	SD	
Diff_ex_	−4.4	28.2	−5.1	36.2	0.47
Diff_comp_	56.6	92.3	30.7	113.4	0.27
Chromogranin A					
Diff_ex_	91.5	122	53.9	65.6	0.13
Diff_comp_	57.9	100.7	39.7	43.4	0.31
Cortisol					
Diff_ex_	33.2	35.7	31.6	30.3	0.46
Diff_comp_	46.2	36.7	56.2	66.7	0.33

**Table 3:** Female group (*n* = 14): diff_ex_(%) and diff_comp_(%) (mean + SD) of for each saliva biomarker in phases 1 and 3.

a-amylase	Phase 1		Phase 3		*p*-value
	Mean	SD	Mean	SD	
Diff_ex_	347.4	583.1	20.3	63.3	<0.05
Diff_comp_	441.8	760.2	27.1	48.8	<0.05
Chromogranin A					
Diff_ex_	53.5	71.1	32.5	29.5	0.11
Diff_comp_	39.3	62.9	27.2	40.4	0.29
Cortisol					
Diff_ex_	29.9	30.4	14.7	35.5	0.12
Diff_comp_	46.6	36.9	29.9	31.2	0.12

### 3.1 α-amylase

Mean Diff_ex_ and Diff_comp_ (% ± SD) for saliva *α*-amylase in Phase 1 were 185.0 ± 457.3 and 264 ± 585.3 respectively, both significantly positive (*p* < 0.05). This suggests that α-Amylase is a stress biomarker since it increased during and immediately after the exercise session compared to baseline values. Mean Diff_ex_ and Diff_comp_ in Phase 1 (see above) were significantly greater than the corresponding figures in Phase 3 (8.6 ± 53.2 and 28.7 ± 83.1 resp, *p* < 0.05) (Table 1).

Mean Diff_ex_ (−4.4 ± 28.2) and Diff_comp_ (56.6 ± 92.3) for men in Phase 1 were no different to the corresponding figures in Phase 3 (−5.1 ± 36.2. *p* = 0.47, and 30.7 ± 113.4. *p* = 0.27 resp) (Table 2).

For the women, mean Diff_ex_ (347.4 ± 583.1) and Diff_comp_ (441.8 ± 760.2) in Phase 1 were significantly different from the corresponding figures in Phase 3 (20.3 ± 63.3 and 27.1 ± 48.8, *P* < 0.05) (Table 3).

### 3.2 Chromogranin A

Mean Diff_ex_ and Diff_comp_ for sCgA in Phase 1 were 71.0 ± 97.7 and 47.9 ± 81.3 respectively, both significantly positive (*p* < 0.05). This again suggests that chromogranin A is an indicator of stress as it increased during and immediately after the exercise session compared to baseline. Mean Diff_ex_ in Phase1 was significantly higher than Diff_ex_ (42.4 ± 49.7) in Phase 3 (*p* < 0.05), whereas mean Diff_comp_ in Phase 1 was not significantly different from mean Diff_comp_ (32.9 ± 41.4) in Phase 3 (*p* = 0.23) (Table 1).

In the male group, mean Diff_ex_ (91.5 ± 122.0) and Diff_comp_ (57.9 ± 100.7) in Phase 1 were not significantly different from Diff_ex_ (53.9 ± 65.6) and Diff_comp_ (39.7 ± 43.4) values in Phase 3 (*p* = 0.13 and *p* = 0.31 for Diff_ex_ and Diff_comp_ respectively) (Table 2).

For the women, mean Diff_ex_ (53.5 ± 71.1) and Diff_comp_ (39.3 ± 62.9) in Phase 1 were not significantly different from mean Diff_ex_ (32.5 ± 29.5) and Diff_comp_ (27.2 ± 40.4) in Phase 3 (*p* = 0.11 and *p* = 0.29 for Diff_ex_ and Diff_comp_ respectively) (Table 3).

### 3.3 Saliva cortisol

Mean Diff_ex_ and Diff_comp_ for saliva cortisol in Phase 1 were 31.4 ± 32.3 and 46.4 ± 36.0 respectively, both significantly positive (*P* < 0.05), indicating that Cortisol is also a stress biomarker as it has increased during and immediately after the exercise session compared to baseline. Mean Diff_ex_ and Diff_comp_ in Phase 1 were not significantly different from Diff_ex_ (22.5 ± 33.7) and Diff_comp_ (42.0 ± 51.4) in Phase 3 (*p* = 0.18 and *p* = 0.36 respectively) (Table 1).

The mean Diff_ex_ (33.2 ± 35.7) for men in Phase 1 was not significantly different from Diff_ex_ (31.6 ± 30.3) in Phase 3 (*p* = 0.46), nor was Diff_comp_ (46.2 ± 36.7) in Phase 1 significantly different from Diff_comp_ (56.2 ± 66.7) in Phase 3 (*p* = 0.33) (Table 2).

Mean Diff_ex_ (29.9 ± 30.4) and Diff_comp_ (46.6 ± 36.9) values for the women in Phase 1 were not significantly different from the corresponding figures in Phase 3 (14.7 ± 35.5 and 29.9 ± 31.2, *p* = 0.12 for both) (Table 3).

## 4 Discussion

The saliva stress biomarkers sAA, sCgA, and sC reliably correlated with mental stress while training on an ERCP simulator. The most accurate prediction of degree of change is obtained by sAA. The response to acute stress involves a complex process which is mediated by the hypothalamic-pituitary-adrenal (HPA) axis, as well as psychological and social reactions.

In the clinical setting, acute stress has a direct impact on performance and patient safety. Endoscopists performing ERCP, frequently encounter highly complex and thus stressful situations. Stress assessment and coping is thus relevant and necessary in this field (2). Thus, the ability to implement a coping strategy to deal with stress is thus important for enhancing performance (11).

Stress is a psychological construct and thus inherently difficult to measure objectively in terms of physiological parameters. Methods assessing autonomic nervous system (ANS) responses in various organ systems have been suggested as surrogate stress markers. These markers include: (a) changes in heart rhythm, measured by heart rate (HR), heart rate variability (HRV), or inter-beat interval (IBI); (b) electrodermal activity (EDA) levels; (c) thermal activity; and (d) saliva stress biomarkers (*i.e.,* sAA, sCgA, sC, and secretory immunoglobulin A). There is a lack of consistent methodology that has led to rather inconclusive and, in certain cases, conflicting results (12).

To our knowledge, this study is the first to use three commercially available saliva stress biomarkers in an ERCP simulation setting. No previous study has reported acute mental strain measurements during endoscopy in clinical and simulation settings (13). In this study, we concomitantly measured saliva a-amylase, saliva chromogranin A, and saliva cortisol, all of them potential stress biomarkers, in a reproducible virtual simulation setting (14–16).

A previous study showed that the biomarkers we used have strong correlations with stress (3). sAA is secreted from the salivary glands but great intra-individual variations are observed (17). It has been suggested that sAA could be used as a surrogate marker of norepinephrine in a variety of stressful conditions (18). In contrast to sC, sAA activity is affected by salivary flow rate and pH (19). Furthermore, in a study stressful situations were associated with higher sAA levels (20).

Chromogranin A (CgA) is a glycoprotein that mediates intracellular storage of catecholamines and is released together with these by the sympathetic nervous system into the blood circulation (21). Previous studies have shown that sCgA responds rapidly to mental stress such as psychosomatic stress [25], academic assessment stress, and computer operation psychological stress (22, 23). Furthermore, it has been observed that sCgA levels increase during mental stress but decrease during intermissions, suggesting that sCgA can be used for short-term assessment of mental workload (24). sCgA is a more accurate indicator than sC since it responds more rapidly and more sensitively to psychological stressors (12). Others, however, have questioned the ability of sCgA to measure stress and/or ANS activity (25).

Plasma cortisol enters the saliva through passive diffusion leading to a stable plasma/saliva ratio. Saliva cortisol levels can thus be used to assess stress related HPA activity, and this has become the most widely used biomarker for studies on mental stress. Saliva cortisol levels begin to rise within 5 min of an increase in plasma cortisol reaching a peak 31–40 min after the onset of the stressor, and saliva levels correlate strongly with plasma cortisol concentrations. Studies have shown that acute stress increases sC levels (3). Some studies have failed to observe such an increase (26) and even a reduction in cortisol level after stress has been reported (11).

The use of saliva stress biomarkers in this study, proved to be simple and without distraction and preferable to invasive methods such as blood sampling (3). Biochemical stress markers were elevated during Phase 1 *i.e.*, during the first attempts to perform ERCP simulator exercises, and decreased as the trainees became acquainted with the simulation environment. This should be considered when planning simulator and clinical training. There were also some differences between the male and female trainees.

As Diff_ex_ and Diff_comp_ in Phase 1 were all significantly positive, we conclude that sAA, sCgA, and sC may be used as stress biomarkers. Of these, the best stress marker appeared to be sAA as it showed the greater percentage increase compared to increases in sCgA and sC (Table 1). It is also evident that familiarisation with the ERCP simulator during Phase 2 led to a reduction in stress while performing exercise in Phase 3, as indicated by the biomarkers used.

Throughout the world, women are highly underrepresented in the field of advanced endoscopy (8). In this study, however, more women participated than men. The stress response appears to be lower in women than in men (27). It has been suggested that this could be caused by a stronger cortisol response in women in the luteal phase than in those in the follicular phase. The menstrual cycle should thus be taken into consideration when assessing the reaction to stress. The cortisol levels may also be affected by oral contraceptives (28). Thus, saliva secretion differs between the sexes, and may be due to variations in the secretion of gonadal steroids and ANS regulation (29).

Our results partially support this observation since sAA Diff_ex_ and Diff_comp_ in the women during Phase 1 were greater than the corresponding values in men. However, sCgA Diff_ex_ and Diff_comp_ were higher in men, and no difference was seen between men and women for sC (Tables 2, 3). When comparing saliva results for sAA, sCgA, sC between Phase 1 and Phase 3, there were no differences in the male group, but sAA showed a statistically significant increase in the female group. Moreover, as can be seen from Tables 2, 3, during Phase 3 both sexes had similar Diff_ex_ and Diff_comp_ for all saliva biomarkers measured (sAA, sCgA, sC).

It should be noted that performance in basic endoscopy does not necessarily correlate with ERCP performance, since no relationship between basic handling skills and therapeutic skills has been demonstrated (30). Furthermore, extrapolation of results from a simulator to the clinical setting should be made with extreme caution. For practical reasons, *in vivo* measurements of physical examination cannot routinely be measured. Endoscopy simulators could, however, play a role in the trainee screening process (31).

In this study, mean sCgA Diff_ex_ and Diff_comp_ during Phase 1 were significantly positive (*p* < 0.05), suggesting that Chromogranin A may be used as a stress biomarker. We also saw that the percentage difference in sCgA between exercise and baseline in Phase 1 was significantly greater than the corresponding difference in Phase 3, indicating that familiarisation in Phase 2 reduced stress in Phase 3.

During Phase 1, mean sC Diff_ex_ and Diff_comp_ were significantly positive (*p* < 0.05), suggesting that saliva cortisol may be used as a stress indicator. This finding concurs with the findings of a study assessing sAA and sC during acute mental stress in 51 surgeons in the OR (25).

This study has some limitations. The sample size was small, and our findings must be interpreted with caution. Secondly, any delayed salivary cortisol response would have been missed and delay in saliva sampling may be necessary to fully detect stress-induced cortisol response (32). No comparison with the FES score was made, and finally, the study focused on simulated ERCP training only, so extrapolation to clinical practice is not feasible. Furthermore, another limitation of the study is that we did not check the oral hygiene of the participants. As it is suggested the latter could influence the accuracy of the saliva biomarkers.

Nevertheless, this study sheds some light on the feasibility of using non-invasive assessment of stress experienced by trainee endoscopists using easy to collect saliva biomarkers. Larger, controlled studies with participants that have different clinical experience is needed to further evaluate and monitor stress during simulation training using saliva biomarkers.

Finally, this was a laboratory study conducted in a controlled environment, whereas stress monitoring in the clinical setting is more complex due to the influence of social, cultural, and psychological factors (33).

## 5 Conclusion

In conclusion, the use of saliva biomarkers for assessing mental stress was easy to implement and well-accepted by all participants in this virtual simulation ERCP training setting. Familiarisation with the ERCP simulator greatly reduced stress when performing ERCP exercises afterwards. The saliva stress biomarkers sAA, sCgA, and sC may all be used to assess mental stress while training on an ERCP simulator, but the best of the three, judging by the degree of change, appears to be sAA. No conclusive difference in stress response between men and women was observed. Further studies including a larger number of trainees with different levels of ERCP experience are needed to exploit performance enhancement and error reduction techniques in ERCP training.

## Data Availability

The raw data supporting the conclusions of this article will be made available by the authors, without undue reservation.
